# Common Mind Troubles and the Secret of a Clear Head

**Published:** 1880-10

**Authors:** 


*Common Mind Troubles and The Secret of a Clear Head.*—By J. Mortimer Granville, M. D., M. R. C. S., etc. Edited with additions by an American Physician. Publisher: D. C. Brinton, Philadelphia, 1880.

The author of this work has quite a reputation in London as an investigator of diseases of the mind, and his treatise has reached the twelfth thousand edition. It is written for popular instruction and contains directions for combating mind troubles which are frequently the warnings of insanity. The contents consist of

PART I. Mental Failings—Defects of Memory—Confusions of Thought—Sleeplessness from Thought—Hesitations in Speech —Low Spirits—Good and Bad Tempers—Mental Languor and Listlessness—Morbid Fears—"Creatures of Circumstances."

PART II. Temperature—Habit—Time—Pleasure—Self-Importance—Consistency—Simplicity—The Secret of a Clear Head.

The work is a very useful one and will repay every one for its closest study.

